# Fast Direct Injection Mass-Spectrometric Characterization of Stimuli for Insect Electrophysiology by Proton Transfer Reaction-Time of Flight Mass-Spectrometry (PTR-ToF-MS)

**DOI:** 10.3390/s120404091

**Published:** 2012-03-27

**Authors:** Marco Tasin, Luca Cappellin, Franco Biasioli

**Affiliations:** 1 IASMA Research and Innovation Centre, Fondazione Edmund Mach, Via E. Mach 1, 38010 San Michele all'Adige, Italy; E-Mail: luca.cappellin@iasma.it; 2 Department of Plant Protection Biology, Swedish University of Agricultural Sciences, P.O. Box 102, 230 53 Alnarp, Sweden; E-Mail: marco.tasin@slu.se; 3 Institute for Ion Physics and Applied Physics, University of Innsbruck, Technikerstr. 25, A-6020, Innsbruck, Austria

**Keywords:** electrophysiological techniques, olfactory stimulus, proton transfer reaction-mass spectrometry, real time monitoring, volatile compounds

## Abstract

Electrophysiological techniques are used in insect neuroscience to measure the response of olfactory neurons to volatile odour stimuli. Widely used systems to deliver an olfactory stimulus to a test insect include airstream guided flow through glass cartridges loaded with a given volatile compound on a sorbent support. Precise measurement of the quantity of compound reaching the sensory organ of the test organism is an urgent task in insect electrophysiology. In this study we evaluated the performances of the recent realised proton transfer reaction-time of flight mass-spectrometry (PTR-ToF-MS) as a fast and selective gas sensor. In particular, we characterised the gas emission from cartridges loaded with a set of volatile compounds belonging to different chemical classes and commonly used in electrophysiological experiments. PTR-ToF-MS allowed a fast monitoring of all investigated compounds with sufficient sensitivity and time resolution. The detection and the quantification of air contaminants and solvent or synthetic standards impurities allowed a precise quantification of the stimulus exiting the cartridge. The outcome of this study was twofold: on one hand we showed that PTR-ToF-MS allows monitoring fast processes with high sensitivity by real time detection of a broad number of compounds; on the other hand we provided a tool to solve an important issue in insect electrophysiology.

## 1. Introduction

Volatile organic compounds are of vital importance in insect life and their detection by olfactory organs has been widely investigated over the last three decades [1,2]. Electrophysiological techniques are used in insect neuroscience to measure the response of olfactory neurons to volatile odour stimuli [3]. Through these studies, scientists may gain information on the functional, structural and morphological organization of the olfactory system, both at the peripheral and at the central level of the olfactory circuit [4]. Along with studies on insect behaviour, knowledge on the olfactory system is essential to understand the basic mechanisms governing the response of insects to volatile olfactory stimuli [5]. Furthermore, research on insect olfaction led to the identification of a number of electrophysiologically and behaviourally active compounds that had a significant impact in the development of safe methods for pest control [6–8].

In electrophysiological recordings, the stimulus is usually delivered to an insect by an air “puff” which conveys volatile molecules from a glass cartridge (or pipette) loaded with a given amount of the test compound, to the insect olfactory organ, *i.e.*, the antennae [9,10]. The simultaneous physiological response elicited by the stimulus on the antennae is recorded by a dedicated hardware [1]. Although this system is very simple to implement, factors such as volatility and affinity of the chemical stimulus to the support and nature of the solvent in which the stimulus is dissolved, make a quantification of the gas cartridge release and thus the result of the experiment elusive. Consequently, drawing conclusions about the relationship between the olfactory stimulus and the physiological response is often a matter of speculation. The solvent in which the compound is dissolved, the dynamic of decay of the stimulus over time (*i.e.*, over puffs) and the chemical purity of both standard odours and air of the laboratory are factors that are expected to introduce further variation to the output of the cartridge. Therefore, a precise measurement of the volatile stimulus exiting the cartridge is expected to provide the actual number of molecules that will be carried over the antennae, allowing an accurate quantification of the effect of the stimulus at a physiological level.

For a proper characterization of the electrophysiological stimuli we need gas sensors that are fast enough to measure gas bursts that last for 1–2 seconds or less; highly sensitive to compete, in the end, with the sensitivity of an insect antenna; highly selective to allow the discrimination of interfering compounds, background gases and the measuring of complex stimuli (gas mixtures). A single technique can hardly fulfil all the requirements. Solid state gas sensors and photoionisation detectors (PID), for instance, are fast but often not enough sensitive and poorly selective, while gas-chromatographic based methods are highly selective but intrinsically too slow. Proton transfer reaction-mass spectrometry (PTR-MS) provides an interesting trade-off between these two opposite situation. It is a direct injection mass spectrometric technique that implements chemical ionisation from H_3_O^+^ ions and has proven to be highly sensitive [11,12]. However, the commercial implementations available so far, based on quadrupole mass analysers that provide unit mass resolution, can measure only few peaks per second with sufficient sensitivity and are too slow for the considered application.

A major breakthrough for the application of PTR-MS in volatile organic compound detection, identification and quantification is the recent introduction [13] and commercialisation [14] of instruments that couple Proton Transfer Reaction ionisation with a Time-of-Flight (ToF) mass analyser (PTR-ToF-MS). This provides a sensitivity that is similar to that of quadrupole based instruments, has larger mass range and, of relevance for this study, and has higher time resolution: with sensitivity in the sub ppb range, a complete mass spectrum up to 400 Th can be recorded in a fraction of a second. At the same time the mass resolution and mass accuracy of PTR-ToF-MS instruments are often enough to define with a high level of confidence the chemical formula of the spectrometric peaks [15]. In this study we employed PTR-ToF-MS as a fast and sensitive gas detector for the characterisation of the electrophysiological stimulus produced by the widespread method of stimulus cartridges.

## 2. Experimental Section

### 2.1. Source of Volatiles

Aliquots of synthetic volatile compounds (see Table 1 for a list) were dissolved in a solvent to obtain a final concentration of 10 μg/μL. With the aim of measuring the effect of the solvent on the stimulus, *n*-hexane (>99.5%; Fluka, Milan, Italy) or paraffin oil (viscid, purissimo; Riedel-de Haën, The Netherlands) were used as solvents. A 5 × 10 mm filter paper loaded with 10 μL of a solution containing a volatile compound was place into a Pasteur pipette (Attrezzature Medico Sanitarie srl, Trento, Italy; specifications: 151 mm total length, 6.8 mm stem diameter, 74 mm stem length, 1.5 mm tip diameter, 50.15 mm tip length), 10 mm inwards from its open end. The opening of the pipette near the filter paper was closed with a 1 mL plastic pipette tip in order to reduce the evaporation of the volatile from the pipette. Pipettes were stored under a hood at 20 °C for 10 minutes before analysis. A Stimulus Controller (Syntech, The Netherlands) with an air flow of 600 mL/min was connected to the opening of the pipette following the removal of the plastic tip. A 2 s stimulus puff was used to push the inner headspace of the pipette into the PTR-ToF-MS detector. Immediately after the puff, the pipette was recapped with the plastic tip and was replaced under hood until the next puff. Pipettes were used for 25 puffs during a 250 min period and then discarded. Each pipette was replicated twice or three times in different days. Five to ten different odours were tested per day. Blank pipettes with only filter paper were used as control. The total number of samples prepared was 950:750 for the decay of the stimulus over time (25 puffs × 5 compounds × 2 solvents × 3 replicates) and 200 for the additional measurements for dose-response experiments (25 puffs × 2 additional concentrations × 2 compounds × 2 replicates). Due to technical faults we lost or disregarded as evident outliers the measurements of 15 samples, most of them in one replicate of pear ester in hexane.

### 2.2. PTR-ToF-MS Analysis

Measurements were performed with a commercial PTR-ToF 8000 [16] instrument (Ionicon Analytik GmbH, Innsbruck, Austria) with the Time of Flight analyser operated in V mode. The sampling time of the ToF spectra is 0.1 ns and the ionisation conditions are controlled by drift voltage (600 V), drift temperature (110 °C) and drift pressure (2.33 mbar), corresponding to an E/N value of about 140 Td. Several spectra are summed in a single spectrum and considered for further analysis every 0.5 second.

The achieved mass resolution was 4,000 (m/Δm_50%_). As compared to quadrupole mass analysers which have unit mass resolution, ToF data analysis is more challenging because of the larger and more complex datasets. A peak extraction procedure is necessary to reduce the number of variables. For this goal we followed the procedure described in [17]. Internal calibration is based on four peaks always present in the PTR-MS spectra: *m/z* = 18.0338 (NH_4_^+^), 21.0221 (H_3_^18^O^+^), 29.9974 (NO^+^) and 59.0491 (C_3_H_7_O^+^). This allows a mass accuracy of better than 0.001 Th (1 Th = 1.036·10^−8^ kg C^−1^) on the relevant region of the spectrum (18–300 Th), as we already pointed out elsewhere [17]. Concentrations are estimated according to Lindinger *et al.* [11] assuming a rate coefficient of 2·10^−9^ cm^3^ s^−1^ for all compounds. This provides, in general, a rough estimation of the concentration which is affected by a systematic error that can be accounted for if the exact reaction coefficient is known [18]. Concentrations are expressed in ppbv (parts per billion by volume). The rationale behind this procedure, and a key point for the presented study, is the fact that for the selected PTR-TOF-MS operating conditions a theoretical derivation of VOC concentration directly from the experimental spectral ion counts expressed in counts per second (cps) is possible without the need of calibrating with a standard [18]. The instrument sensitivity is in fact expressed in cps/ppbv. Using the procedure described in [18] it is possible to estimate the following sensitivities: 20 cps/ppbv for 1-octen-3-ol and 1-octanol, 24 cps/ppbv for methyl salicylate, 27 cps/ppbv for (*E,Z*)-2,4-ethyl-decadienoate and α-humulene. The detection limit is related to the instrumental background, which was less than 10 cps for the considered peaks. The instrumental background converts into a 2σ-limit of detection (LOD) of about 1 ppbv corresponding to 5 μg/m^3^ for 1-octen-3-ol, 1-octanol, methyl salicylate and about 100 pptv (parts per trillion by volume) corresponding to 1 μg/m^3^ for (*E,Z*)-2,4-ethyl-decadienoate and α-humulene. Although we did not measure samples with a defined volume and mass but the fast varying concentration in the plume exiting the pipette, it could be interesting to provide the absolute value of the mass of the compounds entering the instrument. The reported LODs correspond to the injection during the integration time (0.5 s) of less than 15 pg for for 1-octen-3-ol, 1-octanol, methyl salicylate and less than 3 pg for (*E,Z*)-2,4-ethyl-decadienoate and α-humulene. It is worth mentioning that, in general, PTR-TOF-MS detection limit can be improved by increasing the integration time (*i.e.*, the time required for the acquisition of a single spectrum) and can reach values of few pptv [16]. PTR-TOF-MS has a wide linear range that spans from the pptv level to hundreds of ppmv (parts per million by volume), provided effects of detector dead time are properly corrected [19].

In case of saturation of the molecular peak associated to a compound we used the monosubstituted isotopologue for a better estimation of the concentration. Since a continuous controlled flow of air enters the PTR-ToF-MS, the characterisation of the electrophysiological stimulus can be obtained just by inserting the outlet of the cartridges in front of the PTR-ToF-MS inlet and allowing the free diffusion of the excess gas. In order to increase time resolution and reduce memory effects related to the inlet line, we used a short (15 cm) heated (110 °C) peek tube with a inner diameter of 0.25 mm to connect the outlet of the pipette with the drift tube of the PTR-MS, where the ionisation takes place. Before, during and after the gas burst produced by the Stimulus Controller we recorded the PTR-ToF-MS spectra at a 2 Hz rate.

### 2.3. Data Analysis

Spectra were acquired using the software ToF-DAQ (Tofwerk AG, Thun, Switzerland) with a mass range of 0–400 Th. A total of 14,430 spectra were added before storage and only the resulting sum spectra, one each 0.5 s, were stored in HDF5 format (www.hdfgroup.org) and considered for data analysis.

After calibration, correction of detector dead time effects [19] and peak detection, the areas and the maxima of all peaks have been evaluated by Matlab routines. A detailed description of the data analysis methodology can be found in [15]. Further plotting and linear fitting have been performed using R 2.8 [20].

## 3. Results and Discussion

In this study we attempted to characterize stimuli for insect electrophysiology by fast direct injection mass-spectrometry via PTR-ToF-MS. Through the proposed method we achieved an estimate of the concentration of different compounds in the gas exiting the cartridge holding the odorant. The influence of factors such as chemical properties of the odorant, solvent, number of air stimulations and purity of the laboratory air on the output of the cartridge was also measured.

### 3.1. Stimulus Detection by PTR-ToF-MS

**Figure 1.**
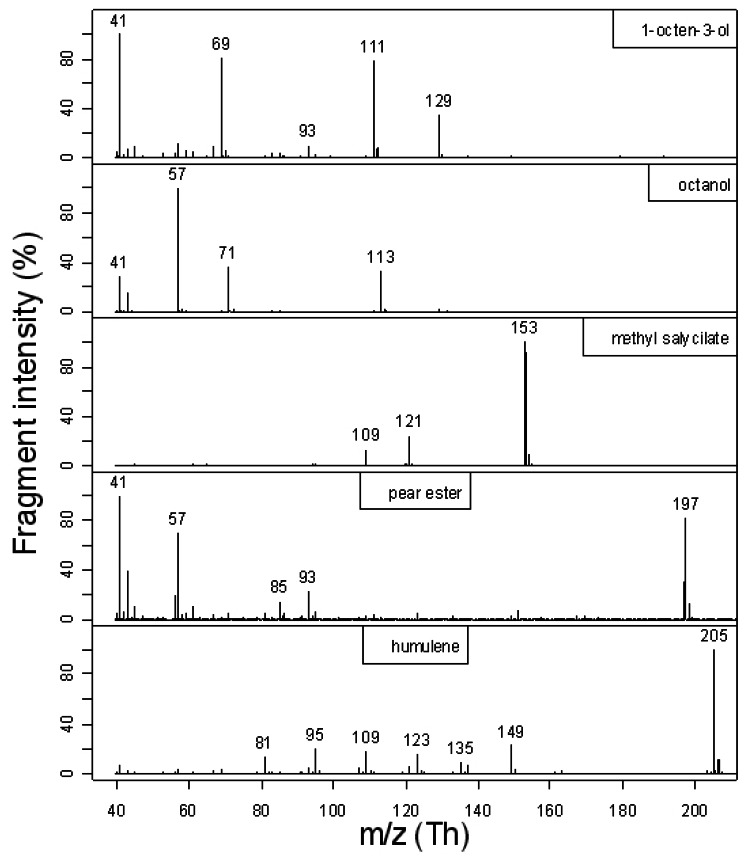
Fragmentation induced by proton transfer to the 5 compounds studied under the experimental conditions used in this work. Intensity is indicated as percentage of base peak (the tallest peak).

Proper compound concentration estimation by PTR-ToF-MS requires a preliminary check of the fragmentation pattern of the compounds considered in our experimental setting. Figure 1 summarises the results. For 1-octen-3-ol we found the same fragments reported by [21], although with differences in intensities probably related to a different E/N value (e.g., more fragmentation at *m/z* = 41). In the case of alcohols, proton transfer is usually accompanied by the loss of a water molecule. However, for 1-octen-3-ol we found, on the contrary of [21], a significant intensity for the molecular peak at *m/z* = 129. For 1-octanol, as expected, we did not observe the molecular peak but only the fragments at *m/z* = 41, 57, 71 and 113. Although the base peak occurs at *m/z* = 57, for the monitoring of 1-octanol we preferred to select the *m/z* = 113 peak in order to avoid limitations due to sensitivity. Moreover, *m/z* = 57 is a common fragment of possible contaminants and it was also present in the fragmentation pattern of the pear ester and, to a lesser extent, of 1-octen-3-ol. Similar arguments hold for the unspecific peaks at *m/z* = 41 and 43. The fragmentation pattern of methyl salicylate and pear ester is, to the best of our knowledge, not available in the literature and it is published here for the first time. The fragmentation of α-humulene is in good agreement with the data of [22]. Based on this evidence we monitored the investigated compounds by measuring the protonated molecular peak or, for alcohols, the protonated molecular peaks after extraction of H_2_O, that is: 111.116 Th for 1-octen-3-ol, 113.132 Th for 1-octanol, 153.054 Th for methyl salicylate, 197.153 Th for pear ester and 205.195 Th for α-humulene. In case of detector saturation [19], we considered the peaks corresponding to their monosubstituted isotopologues.

### 3.2. Time Decay of Stimuli

**Figure 2.**
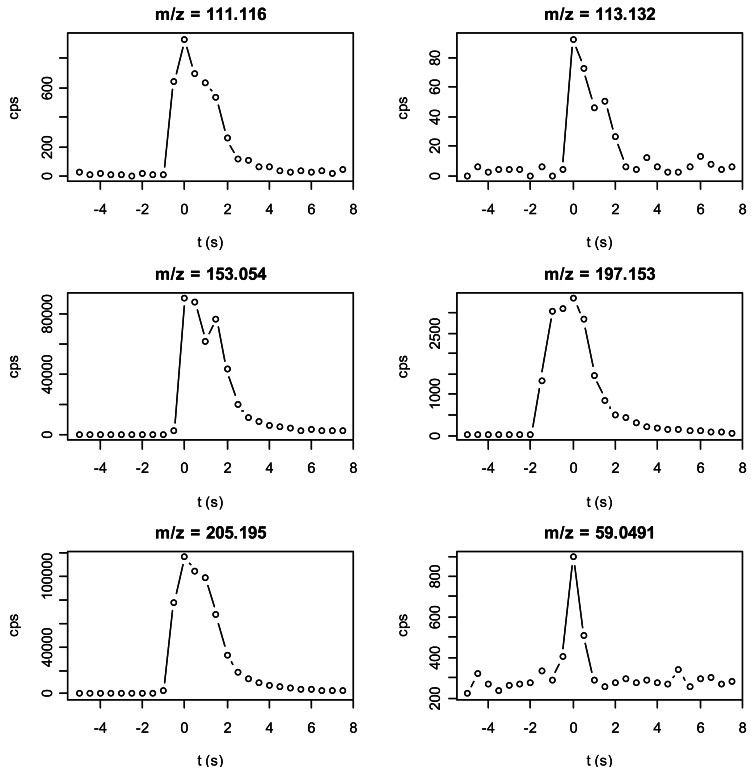
Examples of the time evolution of PTR-ToF-MS signal during gas bursts for the count rates of 5 ions used to monitor the investigated compounds. For each considered compound we show the time evolution of one of its fragments: 111.116 Th for 1-octen-3-ol, 113.132 Th for 1-octanol, 153.054 Th for methyl salycilate, 197.153 Th for pear ester and 205.195 Th for α-humulene. The signal at m/z = 59.049 Th corresponding to acetone (contaminant) is also depicted.

An example of the time evolution of time evolution of concentrations of compounds during a single burst (puff) of the corresponding compound is presented in Figure 2. We had a clear signal for every peak and its width approximately corresponds to the 2 s gas pulse coming from the Stimulus Controller. This indicates that our method is able to follow this rapid dynamic although measuring about 4·10^5^ mass channels in a mass range of more than 400 Th every 0.5 s. We however observed a difference of several orders of magnitude between compounds.

**Figure 3.**
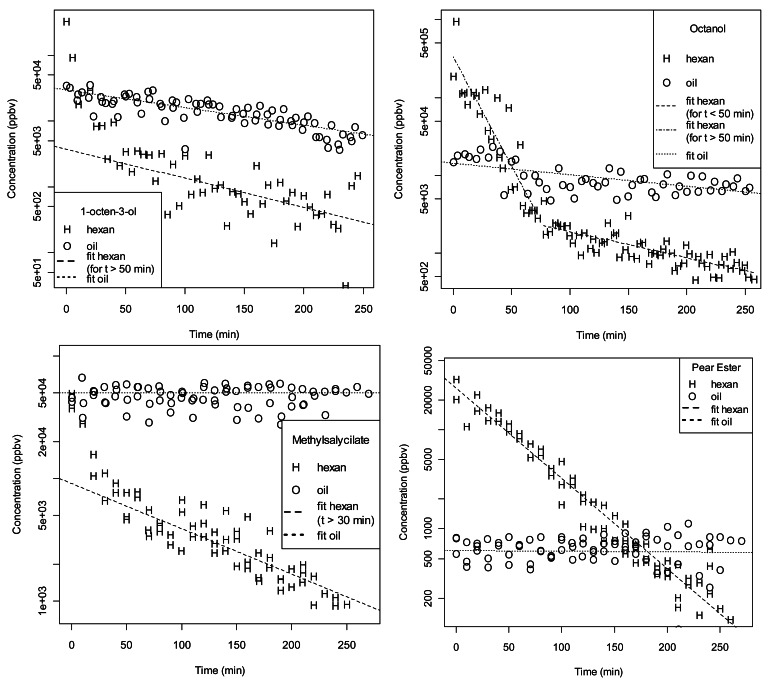
Time evolution (over puffs) of the maximum concentration in the gas emitted by cartridges loaded with a stimulus compound (1-octen-3-ol, 1-octanol, methyl salycilate, or pear ester). The points indicate the solvent: H for hexane and O for paraffin oil. Concentration is estimated from the concentration of the M+1 isotopologue (13.8%). Dashed lines indicate the best fits on the paraffin oil and hexane data separately.

**Figure 4.**
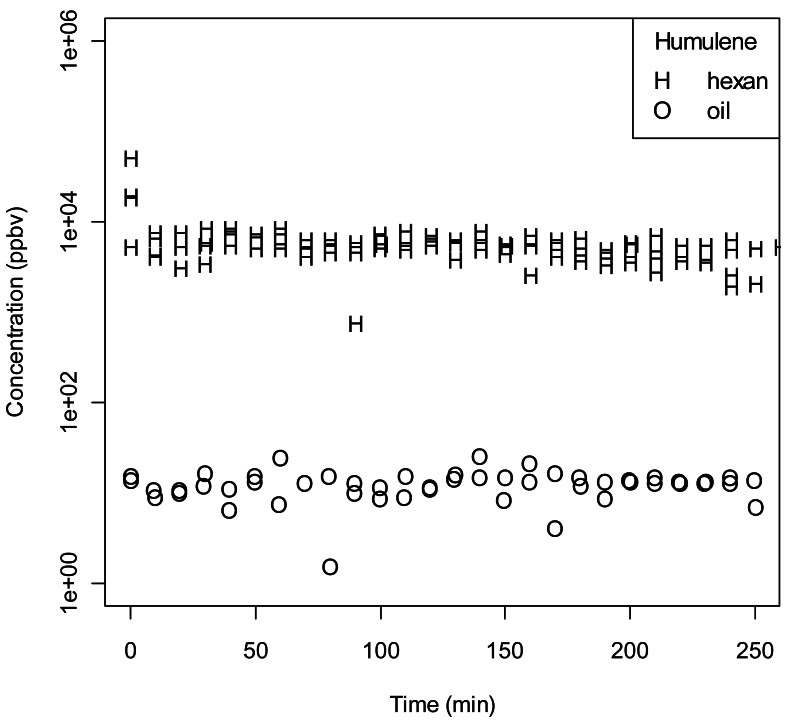
Time evolution for α-humulene.

Figures 3 and 4 depict the time decay of the stimulus, *i.e.*, the maximum signal of the related peak during a burst, over subsequent puffs for all investigated compounds. The starting concentration in the cartridges was always 100 μg. The cases of both hexane and paraffin oil as solvents are presented. The decay of the stimulus over time was strongly affected by the chemical properties of the compound and by the solvent in which the compound was dissolved. A steep decay on the quantity in the puffs was measured when hexane was used as solvent. This decay occurred predominantly within the first puffs for (E,Z)2,4-ethyl-decadienoate, methyl salicylate, 1-octanol and 1-octen-3-ol (Figure 3). A comparable concentration was measured at the first puff (10^5^ ÷ 10^6^ ppbv) for all these compounds. A total decay between 10^2^ and 10^3^ ppbv was measured between the first and the 25th puff. In general the presence of two regimes (rapid exponential decrease for small t followed by a slower exponential decrease) in the hexane data can be explained by the fact that for the first puffs the release of the compound is related to the affinity of the compound for the paper and for the solvent. Instead, for later puffs, hexane is likely to be depleted from the paper and therefore only the partition coefficient (paper/air) of the compound plays a role.

When paraffin oil was used as solvent, a constant quantity in the puffed headspace could be observed (decay < 10 ppbv). In the initial puff the level of molecules strongly differed among compounds: the highest for the two alcohols 1-octanol and 1-octen-3-ol (10^5^ ppbv), the lowest for the monoterpene α-humulene (10^2^ ppbv). A stable release of volatile over time for both solvents was observed for this sesquiterpene (Figure 4). Although constant over time, the amount of this compound in the puffs was a thousand time higher for hexane compared to paraffin oil.

1-Octen-3-ol showed a smooth behaviour when dissolved in paraffin oil. The slow decrease over time (t) of the signal can be described by an exponential function with a time constant of 163 minutes. When hexane was used as solvent, we detected a very rapid decrease in the first 40 minutes followed by a slow decrease with a time constant of 98 min. A similar behaviour was observed for *n*-octanol and methyl salicylate (Figure 3). For the former, the two regimes for hexane are better separated and it is possible to describe them by an exponential decrease also in the region of small t. For 1-octanol in paraffin oil we found a time constant of 297 min in the whole range, while for 1-octanol in hexane we measured a time constant of 15 min for small t and 130 min for larger t. Regarding methyl salicylate, the paraffin oil case does not present any significant decrease in time while the hexane case is described (for t > 30 min) by a time constant of 118 min. A particularly clear pattern is represented by the pear ester, (*E,Z*)-2,4-ethyl-decadienoate (Figure 3). While in the case of paraffin oil we measured a very weak and constant emission of the compound (600 ppbv), in the case of hexane the emission was more intense. The depletion of the compound in the pipette was proportional to its concentration: this led to a exponential decrease of the signal over time. The extrapolated concentration at t = 0 is 26,000 ppbv and the time constant of the exponential is 47.6 minutes. The concentration of α-humulene (Figure 4) does not show any time dependence for both solvents. There is however a remarkable concentration difference: 15 ppb in the case of paraffin oil and 6 ppm in the case of hexane.

**Figure 5.**
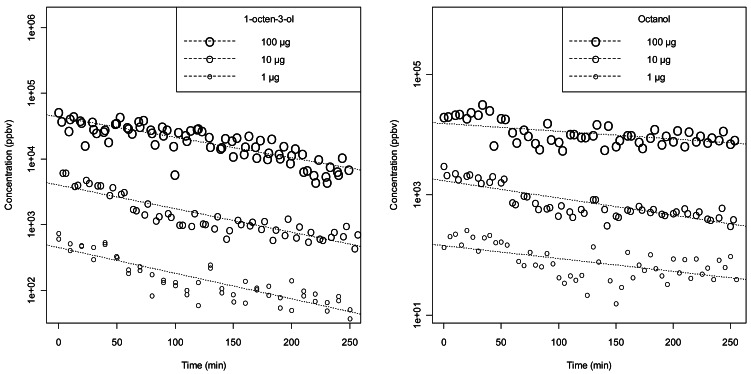
Effect of the starting concentration of the stimulus compound in the cartridges on the detected signal.

Both to verify the linearity of the proposed method and to compare it with typical dose-response experiments on insects, in the case of 1-octanol and 1-octen-3-ol we repeated the measurements with different starting concentrations of compound in the cartridges: 1, 10 and 100 μg/cartridge of compound dissolved in paraffin oil. Figure 5 shows the results and the exponential fits on the data. For 1-octanol we have compatible time dependences (time constant = 190 ± 40 min) and the amplitude of the exponentials are proportional to the initial concentration. The same holds for 1-octen-3-ol with a smaller time constant (125 ± 15 min) and a higher intensity. The total decay was not dependent on the load of the cartridge for both compounds suggesting good linearity and large dynamic range of the proposed method.

### 3.3. Contaminants

**Figure 6.**
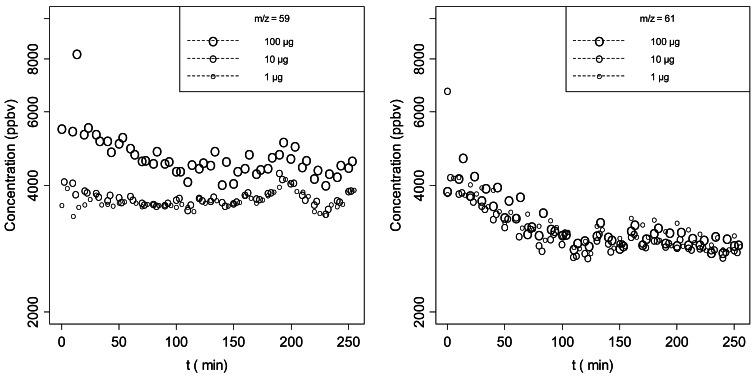
Time evolution of possible contaminants.

As mentioned above, a critical aspect of the application of solid state gas sensors or PID is their lack of selectivity. Therefore, possible contaminants often affect the signal of the selected stimulus compound. On the contrary, PTR-ToF-MS provides enough information for the determination of the chemical formula of the spectrometric peaks and for the simultaneous determination of several peaks. As an example we report (Figure 6) the signal at nominal masses 59 and 61. The peak at *m/z* = 59 corresponds to acetone (propanal contribution is probably negligible) and *m/z* = 61 corresponds to acetic acid and is a fragment of several acetates. This latter fragment is not present in the fragmentation of 1-octanol [21] and its measured concentration is the same even at different concentrations of 1-octanol. However it has a significant signal that, at least in the case of 1-octanol at 1 and 10 μg, can be higher than the signal at *m/z* = 111 used to monitor 1-octanol. It is clear that without selectivity the two signals would overlap thus producing a total signal that would not be a proper estimation of 1-octanol concentration. This can represent a severe drawback for those techniques that do not perform a real time detection of a large number of compounds.

The opportunity to discriminate possible interfering compounds, both from the standard used for preparation or from laboratory air, accounts as a relevant attribute for this kind of investigation: during electrophysiological experiments with odour cartridge, the presence of trace contaminants cannot be excluded and can severely interfere with the response of the test insect. Our results indicate the possibility of accurate stimulus characterisation even in the case of a relatively simple and inexpensive method (pipettes) without the need of a GC column usually applied to partly avoid constraints related to the use of pipettes [23].

## 4. Conclusions

In this study we employed fast direct injection Proton Transfer Reaction–Time-of-Flight Mass Spectrometry (PTR-ToF-MS) to characterise the outcome of cartridges loaded with a set of volatile compounds belonging to different chemical classes and commonly used in electrophysiological experiments. Through this study we demonstrate that PTR-ToF-MS is a sensitive and highly specific sensor that can follow rapid processes. At the same time, this instrument can provide an accurate characterisation of the actual stimulus (*i.e.*, number of molecules) conveyed to insect antennae during electrophysiological experiments.

PTR-ToF-MS allowed a fast monitoring of all investigated compounds with sufficient sensitivity and time resolution. In our experimental conditions, the decay of the stimulus over time was strongly affected by the chemical properties of the compound and by the solvent in which the compound was dissolved. For all the tested compounds except α-humulene, a steep decrease in the level of molecules in the headspace was observed already after a few puffs when hexane was used as solvent. Therefore, the use of hexane in electrophysiological studies needs to be carefully evaluated, due to the variability of the stimulus over time. On the other hand, a stable and more reliable outcome was measured when paraffin oil was used as solvent. This solvent compared to hexane significantly reduced the decay over time. From these results, it appears that paraffin oil may be the recommended solvent to be employed when preparing solution for electrophysiological recordings. Nevertheless, for extremely volatile compounds with a low solubility in paraffinic oil, the use of other solvents such as hexane or pentane may still be recommended, along with a precise measurement of the decay of the stimulus over time. Another relevant result of our study is the simultaneous detection and quantification of contaminants, both from the laboratory atmosphere and from the solvent or the synthetic standard used as stimulus. Although the outcome of our study remained to be correlated with physiological outputs and validated over a larger number of compounds, high sensitivity (ppb-level) combined with a real time detection of a broad number of compounds makes PTR-ToF-MS a promising technique for studies dealing with the quantification of odour stimuli for insect electrophysiology.

## Figures and Tables

**Table 1.** List of compounds used as stimuli in the experiments and their chemical properties.

**Compound**	**Chemical class**	**Formula**	**Molecular weight (Da)**	**Boiling point (°C at 760 mmHg)**	**Density (g/cm^3^)**	**CAS number**	**Source of standard**	**Purity (%)**	**Experiments**		

									**Solvent**		

									**Paraffin oil**	**Hexane**	**Paraffin oil (dose response)**
1-octen-3-ol^a^	aliphatic monounsaturated alcohol	C_8_H_16_O	128.21	174	0.837	3391-86-4	Acros	98	x	x	x
1-octanol^a^^,^^b^^,^^c^	aliphatic alcohol	C_8_H_18_O	130.23	195	0.824	111-87-5	Acros	98	x	x	x
methyl salicylate^a^^,^^b^^,^^d^	benzenoid	C_8_H_8_O_3_	152.15	220	1.174	119-36-8	Sigma-Aldrich	99	x	x	-
(E,Z)2,4-ethyl-decadienoate^a^^,^^d^	diunsaturated ester	C_10_H_20_O_2_	196.29	246	0.903	3025-30-7	Fluka	97	x	x	-
α-humulene^a^^,^^c^^,^^d^	sesquiterpene hydrocarbon	C_15_H_24_	204.36	107	0.886	6753-98-6	Sigma-Aldrich	98	x	x	-

a attractant;

b pheromone;

c allomone;

d kairomone
